# Cranberry Polyphenols and Prevention against Urinary Tract Infections: New Findings Related to the Integrity and Functionality of Intestinal and Urinary Barriers

**DOI:** 10.1021/acs.jafc.3c07169

**Published:** 2024-04-23

**Authors:** Dolores González de Llano, Mikel Roldán, Diego Taladrid, Edgard Relaño de la Guía, M. Victoria Moreno-Arribas, Begoña Bartolomé

**Affiliations:** Institute of Food Science Research (CIAL), CSIC-UAM, C/Nicolás Cabrera 9, Madrid 28049, Spain

**Keywords:** cranberry, urinary tract infections (UTI), phenolic metabolites, intestinal barrier, urinary barrier, TEER, paracellular permeability, tight junctions

## Abstract

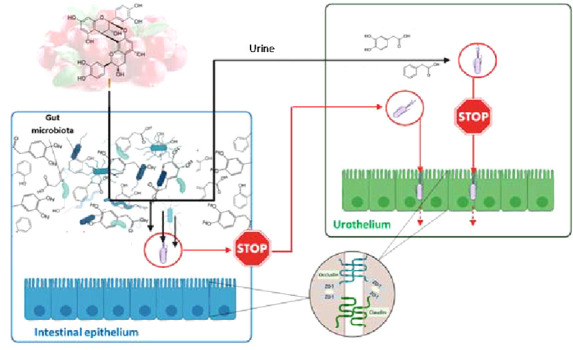

This work seeks to generate new knowledge about the mechanisms underlying the protective effects of cranberry against urinary tract infections (UTI). Using Caco-2 cells grown in Transwell inserts as an intestinal barrier model, we found that a cranberry-derived digestive fluid (containing 135 ± 5 mg of phenolic compounds/L) increased transepithelial electrical resistance with respect to control (ΔTEER = 54.5 Ω cm^2^) and decreased FITC-dextran paracellular transport by about 30%, which was related to the upregulation of the gene expression of tight junction (TJ) proteins (i.e., occludin, zonula occludens-1 [ZO-1], and claudin-2) (∼3–4-fold change with respect to control for claudin-2 and ∼2–3-fold for occludin and ZO-1). Similar protective effects, albeit to a lesser extent, were observed when Caco-2 cells were previously infected with uropathogenic *Escherichia coli* (UPEC). In a urinary barrier model comprising T24 cells grown in Transwell inserts and either noninfected or UPEC-infected, treatments with the cranberry-derived phenolic metabolites 3,4-dihydroxyphenylacetic acid (DOPAC) and phenylacetic acid (PAA) (250 μM) also promoted favorable changes in barrier integrity and permeability. In this line, incubation of noninfected T24 cells with these metabolites induced positive regulatory effects on claudin-2 and ZO-1 expression (∼3.5- and ∼2-fold change with respect to control for DOPAC and ∼1.5- and >2-fold change with respect to control for PAA, respectively). Overall, these results suggest that the protective action of cranberry polyphenols against UTI might involve molecular mechanisms related to the integrity and functionality of the urothelium and intestinal epithelium.

## Introduction

Urinary tract infections (UTI) are the most pervasive type of bacterial infection, mainly affecting the female population and resulting in huge healthcare costs worldwide.^1^ Specifically, bacterial infections of the bladder (cystitis) and associated structures (lower UTI) are the most common uncomplicated UTI. However, several risk factors can lead to treatment failure, recurrent infections, and significant morbidity and mortality with poor outcomes. Moreover, complicated UTI are among the most common causes of sepsis cases presenting to hospitals.^2^ Among the common uropathogens associated with these infections, uropathogenic *Escherichia coli* (UPEC) is the primary cause.^3^ Conventional therapies against UTI have been largely based on antibiotics, which seem to increase the prevalence of multidrug-resistant uropathogens and generate adverse side effects on the intestinal microbiota, not to mention increased recurrence rates.^4^ Microbial host-associated reservoirs in the underlying bladder tissue or gastrointestinal tract could cause reinfection even after intensive treatment and subsequent negative urine culture.^5^

Urothelium dysfunctions have been closely associated with UTI pathogenesis and/or its recurrence,^6^ whereas the cross talk between intestinal barrier dysfunction and UTI has not been extensively studied.^7^ Both the intestinal and urinary tracts are lined with layers of epithelial cells that provide permeability to allow a rapid exchange of solutes and nutrients, and these layers also maintain a physical and functional barrier to harmful microorganisms and their products. Preservation of the integrity and functionality of the intestinal barrier is dependent on the intactness of the apical plasma membrane on the epithelial cells as well as the intercellular tight junctions (TJ).^8^ Moreover, TJ disruption can cause increased intestinal permeability, leading to “leakiness” in the way that pathogens (i.e., extraintestinal pathogenic *Escherichia coli* [ExPEC]) can cross the gut epithelium via paracellular permeation, facilitating their translocation to the urinary tract.^7^ In the case of the bladder, it has been reported that the urothelium permeability increases as the expression of TJ proteins decreases, allowing the entry/passage of bacteria as well as the passage of ions through the blood-urinary barrier.^9^ In addition, it has been found that UPEC infection disrupts the tight binding barrier with decreased expression of TJ proteins.^10^ Therefore, any strategy designed to strengthen the urothelial barrier would decrease the risk of urinary tract infection.^11^

Consuming cranberry (*Vaccinium macrocarpon*) has been reported as a nonantibiotic prophylactic and therapeutic alternative for recurrent UTI.^12^ Cranberry stands out for its high content of polyphenols, mainly proanthocyanidins, anthocyanidins, and flavonols,^13,14^ which undergo extensive metabolism during their colonic digestion, triggering a wide range of phenolic metabolites, mainly benzoic, hydroxycinnamic, phenylpropionic, and phenylacetic acids, as well as microbial intermediate metabolites such as phenylvaleric acids and phenyl-γ-valerolactones.^15,16^ These microbial-derived phenolic metabolites are known to be present in urine after cranberry intake^17−19^ and could be responsible, at least partially, for its beneficial effect due to their antiadhesive activity against uropathogens (i.e., UPEC).^20−22^ On the other hand, as it is becoming evident that the intestine is a reservoir for uropathogenic bacteria, *in vitro* studies have indicated that cranberry flavonoids and phenolic acids might interact with ExPEC and decrease its (transient) intestinal colonization, consequently reducing the risk of UTI incidence.^23^ More recently, and using an original disposition of a well-known dynamic simulator (SHIME) of the digestive tract, Roussel et al.^24^ demonstrated that microbial-derived proanthocyanidin metabolites exhibited a significant blunt activation of UPEC virulence genes at an early stage in the gut reservoir, which affects infectivity on the urothelium in a microbiota-dependent manner, thereby explaining the preventative contentious properties of cranberry against UTI. In relation to the intestinal epithelial barrier, Faggian et al.^25^ carried out a pioneering study demonstrating that cranberry extracts improved barrier functionality in Caco-2 cells and inhibited the production of inflammatory cytokines, both at baseline and under stress conditions (H_2_O_2_ or ExPEC). However, this work presented a certain limitation, as using the extracts directly, without a previous intestinal digestion, ignored the fact that polyphenols are extensively metabolized during their passage through the gastrointestinal tract.

With the ultimate aim of generating new knowledge about the mechanisms underlying the protective effects of cranberry against UTI, the effects of cranberry-derived phenolic metabolites on the integrity and functionality of intestinal and urinary barriers were investigated. For that, we used two *in vitro* epithelium models (Caco-2 and T24 cells) that were either noninfected or infected with a UPEC strain. For the intestinal epithelium model, Caco-2 cells were incubated with the cranberry-derived digestive fluid obtained from the *in vitro* digestion of a polyphenol-rich cranberry extract in the dynamic gastrointestinal simulator simgi (CIAL-CSIC, Madrid, Spain).^16^ For the urothelium model, T24 cells were incubated with 3,4-dihydroxyphenylacetic acid (DOPAC) and phenylacetic acid (PAA), two of the cranberry-derived phenolic metabolites widely tested in studies investigating the molecular mechanisms behind the protective effects of cranberry against UTI.^1^ Changes in barrier integrity were measured as transepithelial electrical resistance (ΔTEER), whereas changes in barrier functionality were measured as paracellular transport with fluorescein isothiocyanate (FITC)-dextran and as TJ proteins [i.e., occludin, zonula occludens-1 (ZO-1), and claudin-2] expression.

## Materials and Methods

### Chemicals

Two phenolic compounds were tested in this study, namely, 3,4-dihydroxyphenylacetic acid (DOPAC) (≥97%) and phenylacetic acid (PAA) (≥99%), both purchased from Sigma-Aldrich (St Louis, MO). Both compounds have been found in urine after cranberry intake, together with other main phenolic metabolites.^17^

### Samples

A cranberry-derived digestive fluid was prepared from a cranberry extract provided by Ocean Spray Cranberries, Inc. (Middleborough, USA) and previously characterized with a view to using it in the experiments concerning the intestinal barrier.^16^*In vitro* digestion of the extract, including gastrointestinal digestion and colonic fermentation, was carried out in a dynamic gastrointestinal simulator (simgi, CIAL-CSIC, Spain) as described in Tamargo et al.^16^ The simgi consists of five compartments (stomach, small intestine, ascending colon, transverse colon, and descending colon), which enable simulation of the different stages of gastrointestinal digestion and colonic fermentation.^26,27^ Once the fecal microbiota had been stabilized in the three colonic reactors, the system was fed (chronic feeding) with 80 mL of a fresh solution of cranberry extract (4.17 mg/mL) in nutrient medium three times per day (every 8 h), which supposed a daily feeding of 1 g of extract.^16^ For this study, effluents from simgi (after the whole digestion process) were collected just before cranberry feeding (sample named “Ef”) and after 15 days of cranberry feeding (sample named “CB-ef”). Immediately after collection, the samples were centrifuged (10,000 rpm, 10 min, 4 °C). Supernatants were filtered through a 0.22 μm membrane, aliquoted, and stored at −20 °C for further use. The phenolic composition of both effluents was determined by UPLC-ESI-MS/MS (Table 1).

**Table 1:** Phenolic Composition in the simgi Effluents Ef and CB-ef

compound	concentration (mg/L)	
benzoic acids	CB-ef	Ef
3-hydroxybenzoic acid	0.083 ± 0.004	traces
4-hydroxybenzoic acid	0.614 ± 0.040	0.252 ± 0.011
3,4-dihydroxybenzoic acid (protocatechuic acid)	0.130 ± 0.004	traces
2-hydroxybenzoic acid (salicylic)	0.241 ± 0.007	0.0172 ± 0.0040
4-hydroxy-3-methoxybenzoic acid (vanillic acid)	4.11 ± 0.285	traces
3,5-dimethoxy-4-hydroxybenzoic acid (syringic acid)	0.122 ± 0.015	nd
benzoic acid	70.7 ± 1.6	0.601 ± 0.025
benzene-1,2-dicarboxylic acid (phthalic acid)	traces	0.0901 ± 0.0012
phenylacetic acids		
3-hydroxyphenylacetic acid	8.17 ± 0.22	nd
4-hydroxyphenylacetic acid	2.43 ± 0.21	4.27 ± 0.59
3,4-dimethoxyphenylacetic acid	2.47 ± 0.08	nd
4-hydroxy-3-methoxyphenylacetic acid (homovanillic acid)	0.716 ± 0.020	traces
phenylacetic acid	14.5 ± 0.86	31.9 ± 1.8
phenylpropanoic acids		
3-(3′-hydroxyphenyl)propanoic acid	4.15 ± 0.13	0.796 ± 0.073
3-(4′-hydroxyphenyl)propanoic acid	17.5 ± 1.5	traces
cinnamic acids		
4′-hydroxycinammic acid (*p*-coumaric acid)	0.225 ± 0.013	traces
mandelic acids		
4-hydroxy-3-methoxymandelic acid	0.0300 ± 0.0020	nd
stilbenes		
dihydro-piceid	0.0301 ± 0.0061	nd
valerolactones		
γ-valerolactone	0.0172 ± 0.0052	0.0150 ± 0.0030
valeric acids		
phenyl-4-hydroxyvaleric acid	4.40 ± 0.13	nd
hydroxyphenyl-4-hydroxyvaleric acid	0.0123 ± 0.0031	nd
flavan-3-ols		
procyanidin A2	4.15 ± 0.13	nd
		
total compounds	135 ± 5	38.1 ± 2.5

Urine from a healthy young male volunteer was collected during 24 h. The volunteer had received no antibiotics, probiotic treatment, vitamin supplements, or any other medical treatment in the 12 months before the experiment. Urine was collected after a 2 day strict diet lacking polyphenol-containing foods (i.e., without vegetables, fruits, coffee, and tea, among others). The volunteer signed an informed consent form, and the study was performed in compliance with the Declaration of Helsinki. Once homogenized, the urine was filtered through a 0.22 μm membrane, aliquoted, and stored at −20 °C for further use.

### Human Cells and Bacteria

Human epithelial colorectal adenocarcinoma cells (Caco-2 cells, ATCC HTB-37TM) and human epithelial bladder cells (T24 cells, ATCC HTB-4) were selected as cellular models of intestinal and urothelial barriers, respectively. Caco-2 cells grown on filter supports have been the preferred model of the intestinal epithelium in recent decades, as these cells are able to differentiate spontaneously into a polarized cell layer with apical microvilli upon reaching confluence, providing many of the properties associated with the enterocytes of the small intestine.^28^ Although there is no proper biomimetic human culture model to generate a functional urothelial barrier *ex vivo*, *in vitro* propagated urothelial or bladder cells have been used to simulate urothelium.^29,30^ This is the case for the T24 human epithelial bladder cells used in this study.

Caco-2 cells (passage range of 25–35) were cultured in high-glucose Dulbecco’s modified Eagle’s medium (DMEM) (Biowest, Nuaillé, France) supplemented with a 1% (v/v) penicillin/streptomycin solution (Sigma-Aldrich, St. Louis, MO), a 10% (v/v) heat-inactivated fetal bovine serum (FBS) solution (Biowest, Nuaillé, France), and a 1% (v/v) nonessential amino acid solution (Lonza, Basel, Switzerland). T24 cells (passage range of 15–20) were cultured in McCoy’s 5A medium with l-glutamine (Lonza, Basel, Switzerland) supplemented with a 1% (v/v) penicillin/streptomycin solution and a 10% (v/v) FBS solution. Both cell lines were cultured at 37 °C in a 5% CO_2_ atmosphere with constant humidity.

A uropathogenic *E. coli* (UPEC) strain from the American Type Culture Collection (*E. coli* ATCC 53503) that expresses P fimbriae^31^ was used. To preserve this microorganism, vial aliquots in 20% glycerol were initially prepared and stored at −70 °C. UPEC was grown in a liquid culture medium (tryptic soy broth, TSB) (Scharlau, Barcelona, Spain) at 37 °C and agitated for 16 h, and colony forming units (CFUs) were quantified in tryptic soy agar (TSA) by dilution plating. UPEC inocula were prepared by centrifuging (10,000*g*, 10 min, 4 °C) overnight cultures and resuspending them in cell culture media (DEMEN or McCoy’s 5A) at concentrations of about 10^8^ CFU/mL.

### Cytotoxicity Assays

For Caco-2 cytotoxicity, simgi effluents (CB-ef and Ef) were diluted 1:50 (v/v) with Caco-2-cell growth medium without FBS. This dilution range was established from previous data on the cell cytotoxicity of the effluents.^16^ For the phenolic metabolite DOPAC, a stock solution was prepared in DPBS (Dulbecco’s phosphate-buffered saline, Sigma-Aldrich, St Louis, MO) at a concentration of 1 mM and was then stored at −20 °C for further use. The DOPAC solution to be tested was at a concentration of 250 μM by diluting the stock solution with Caco-2-cell growth medium without FBS. For T24 cytotoxicity, urine was nondiluted or diluted 1:2 and 1:4 (v/v, urine volume/final volume) with T24 cell growth medium. As 1:4 diluted urine was the only one that showed no toxicity against T24 cells, phenolic standards (DOPAC and PAA) were prepared in the urine/medium mixture (1:4 v/v) at a concentration of 250 μM. All assayed solutions were filtered through a 0.22 μm filter (Symta, Madrid, Spain) just prior to use.

The cellular toxicity against Caco-2 and T24 cells was measured using the colorimetric 3-(4,5-dimethylthiazol-2-yl)-2,5-diphenyltetrazolium bromide (MTT) assay. In brief, Caco-2 or T24 cells, seeded the previous day at a density of 5 × 10^5^ cells/mL (100 μL/well) on 96-well plates, were incubated with the respective cell culture medium (control) or with test samples for 24 h at 37 °C in a 5% CO_2_ atmosphere and constant humidity. Then, the supernatants were replaced by the MTT reagent (Sigma-Aldrich, St. Louis, MO) (0.5 mg/mL). After 3 h of incubation, the MTT reagent was removed from the wells, and 100 μL of dimethyl sulfoxide (DMSO, Sigma-Aldrich, St. Louis, MO) was added to dissolve formazan crystals. Absorbance at 570 nm was measured on a Multiskan plate reader (Thermo Fisher Scientific, Portsmouth, NH). Control absorbance was considered to be 100% cell viability, and the results were expressed as the percentage of cell viability relative to untreated control cells. Assays were carried out in triplicate, and three independent experiments were performed.

### Cell Culture Experiments

**Figure 1:**
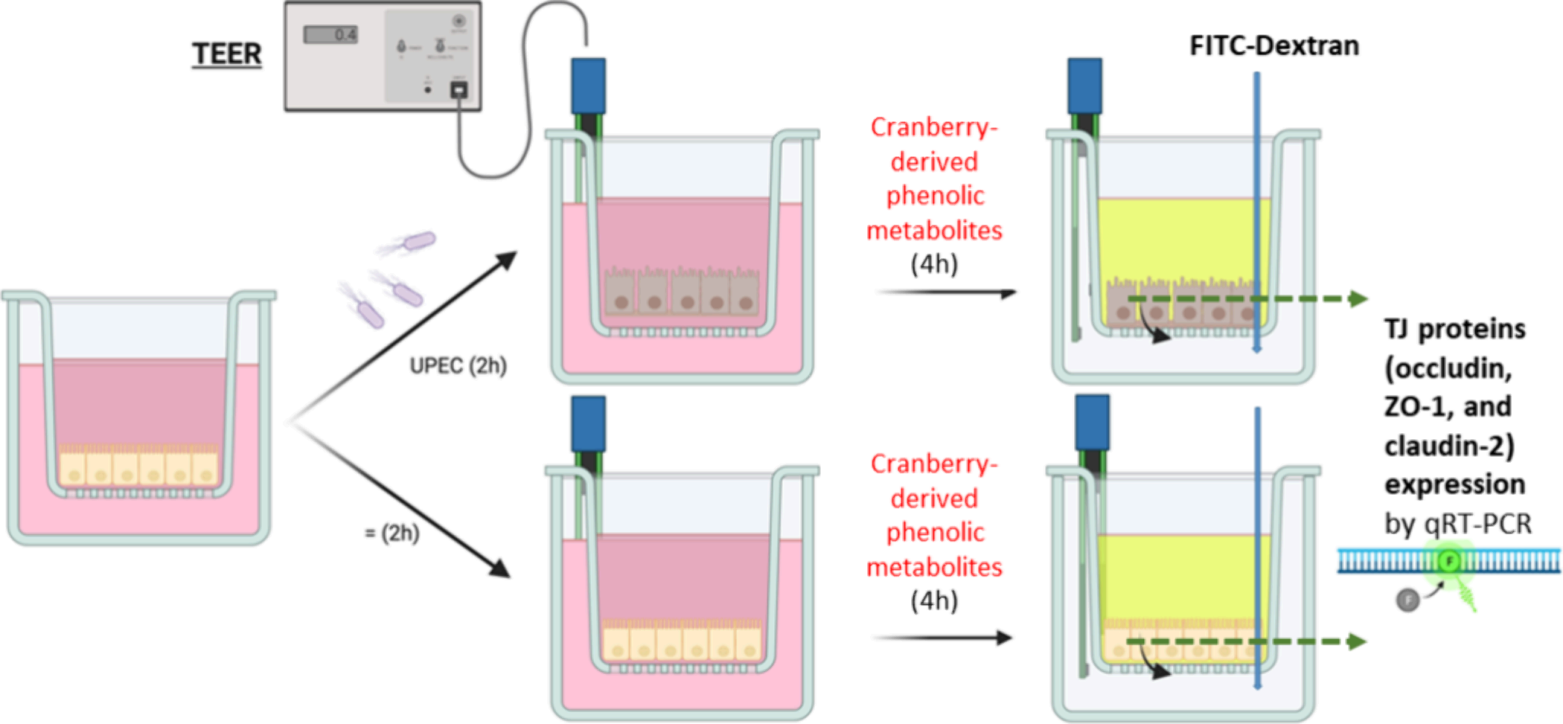
Schema of experiments on Transwell inserts for both Caco-2 and T24 cells. Changes in barrier integrity were measured as transepithelial electrical resistance (TEER). Changes in barrier functionality were measured as paracellular transport with FITC-dextran and as TJ protein (occludin, ZO-1, and claudin-2) expression.

Cell culture experiments were designed to assess the effects of cranberry-derived phenolic metabolites on the integrity and functionality of both intestinal and urinary barriers (Figure 1). Caco-2 cells grown at confluence were seeded at 1.5 × 10^5^ cells/mL (2 mL/well) in polycarbonate Transwell inserts (24 mm Ø, 0.4 μm pore size) (Costar, Corning Incorporated, Kennebunk, USA) and cultured as indicated above. After renewing the medium every 3 days, transepithelial electrical resistance (TEER) was measured with an Epithelial Volt/Ohm Meter (World Precision Instruments, Sarasota, USA) to ensure that the cells had reached confluence and differentiation, which occurred at 21 days. M6 Polycarbonate Transwell inserts were also used for the experiments with T24 cells. They were seeded at 5 × 10^5^ cells/mL (2 mL/well) and cultured for 3 days to enable cell attachment and to obtain confluent cell monolayers. Also, TEER was measured after renewing the medium every day. Then, to simulate a healthy state or an infection by UPEC, the polarized Caco-2 or confluent T24 cell monolayers were washed with DPBS to eliminate antibiotic residues and overlaid with 1.5 mL of either cell culture medium (as the healthy state control) or UPEC inocula (cell-to-bacteria ratio of 1:100). After 2 h at 37 °C in a 5% CO_2_ atmosphere, infected monolayers were gently washed with DPBS to remove unbound bacteria and incubated again with the cell culture medium (2 mL/well). To evaluate the effect of UPEC on barrier integrity, the TEER of each insert (incubated or not incubated with UPEC) was measured (TEER_(*t*=0h)_).

Afterward, the apical medium of the Transwell inserts containing the Caco-2 cell monolayers was removed, and 2 mL of the DMEM (as control) or of each sample (CB-ef and Ef [1:50; v/v] or 250 μM DOPAC, diluted in DMEM) was added to duplicate inserts, noninfected (−UPEC) or infected (+UPEC) with the uropathogenic bacteria. In the T24 cell monolayers, in a similar way, the apical medium was replaced by adding 2 mL of McCoy’s 5A medium (as control) or samples (urine diluted 1:4 [v/v], and DOPAC and PAA [250 μM] diluted in urine/medium [1:4] [v/v]). After that, and for both experiments with Caco-2 cells and T24 cells, plates were incubated at 37 °C for 4 h, simulating the time during which dietary compounds remain in the small intestine under *in vivo* conditions. Then, a new measurement of the TEER was carried out to evaluate their bioactive effects on barrier integrity (TEER_(*t*=4h)_). The final TEER values of the samples were expressed as ΔTEER_sample_ with respect to the control to normalize the TEER and correct calibration errors and instrumental variations. Therefore, given that ΔTEER_control_ = TEER_(*t*=4h)control_ – TEER_(*t*=0h)control_, the final expression was ΔTEER_sample_ (with respect to the control) = (TEER_(*t*=4h)sample_ – TEER_(*t*=0h)sample_) – (TEER_(*t*=4h)control_ – TEER_(*t*=0h)control_).

In addition, paracellular transport with 4 kDa fluorescein isothiocyanate (FITC)-dextran (Sigma-Aldrich, St. Louis, MO) was measured. When the incubation time with cranberry-derived phenolic metabolites was over, the apical chamber solutions of the Transwell culture inserts were replaced by 2 mL of a DPBS solution containing 1 mg/mL of FITC-dextran, and the basolateral sides of the plate inserts were filled with DPBS. Then, the plate was incubated at 37 °C for 30 min. Afterward, 100 μL from the basolateral chamber of each well was taken in triplicate to measure the concentration of FITC-dextran by fluorescence intensity in a BioTek FL600 microplate reader (BioTek, Winooski, USA) with an excitation wavelength of 480 nm and an emission wavelength of 520 nm. Results were expressed as % of permeability with respect to the control. For both cell lines and for each cell culture experiment, incubations in Transwell inserts were carried out in triplicate, and experiments were repeated on 3 different days.

### TJ Protein Expression Assay

At the end of the cell culture experiments, Caco-2 or T24 cell monolayers from each Transwell insert were washed with DPBS. Then, they were scraped and withdrawn with 750 μL of cold DPBS and further centrifuged at 1500 rpm for 10 min. Pellets were resuspended in 350 μL of the RA-1 buffer (with 1% β-mercaptoethanol) (Sigma-Aldrich, St. Louis, MO) by using the vortex and then stored at −70 °C until RNA extraction. A NucleoSpin RNA XS kit (Macherey-Nagel, Düren, Germany) was used for RNA extraction following the manufacturer’s instructions. cDNA was obtained with a qPCRBIO cDNA Synthesis kit (PCR Biosystems, Wayne, USA) according to the procedure described by the manufacturer. Data on the quantity (ng/μL) and quality assurance (Ratio 260/280) of extracted RNA are reported in Table S1.

Finally, qRT-PCR was performed in a ViiA 7 Real-Time PCR system (Applied Biosystems, Foster City, USA). Quantification of genetic expression of TJ proteins (occludin, ZO-1, and claudin-2) by qRT-PCR was carried out using the GA3PDP (glyceraldehyde 3-phosphate dehydrogenase) gene as housekeeping as previously described.^32,33^ The primers employed in the amplification and annealing temperature are reported in the Supporting Information (Table S2). The concentration of each primer was previously optimized. An initial 0,5 uM solution was serially diluted down to 1:1000 (v/v). After analysis, the 1:10 dilution was established as the proper one. Melting curves showed single peaks for each pair of primers, which ensured the specificity of the amplification products. The mRNA levels of each protein in the different samples were normalized against the GA3PDPH gene and expressed as the fold increase with respect to their respective control using the *E^–^*^ΔΔCT^ method.

### Determination of Phenolic Composition

Aliquots of simgi effluents (Ef and CB-ef) were defrosted and filtered through 0.22 μm PVDF filters (Symta, Spain). Then, 200 μL of samples were mixed with 50 μL of internal standard (4-hydroxybenzoic-2,3,5,6-tetradeutered acid) (IS) prepared in acetonitrile and 0.1% formic acid at a final concentration of 0.25 mg/mL. Analysis of phenolic compounds was carried out by UHPLC-DAD-ESI-TQ MS following a previously reported method.^34,35^ The liquid chromatographic system was a Waters Acquity UPLC (Milford, MA, USA) composed of a binary pump, a thermostated autosampler, a heated column compartment, a photodiode array detector, and an Acquity TQD tandem quadrupole mass spectrometer equipped with a Z-spray electrospray ionization (ESI) source. Chromatographic conditions and detection parameters were previously optimized,^34,35^ with 2.0 μL of each sample being injected into the chromatographic system. Briefly, the column employed was a BEH-C18, 2.1 × 100 mm and 1.7 μm particle size from Waters (USA). The gradient consisted of A (water/formic acid; 90:10, v/v) and B (acetonitrile) applied as follows: 0–1 min, 5–15% B; 1–5.25 min, 15–24% B; 5.25 to 5.88 min, 24–100% B; 5.88 to 7.05 min, 100–5% B; and 7.05 to 9.38 min, 5% B. The flow rate was set constant at 0.5 mL/min. The ESI parameters were set as follows: capillary voltage, 3 kV; source temperature, 130 °C; desolvation temperature, 400 °C; desolvation gas (N2) flow rate, 750 L/h; and cone gas (N2) flow rate, 60 L/h. Phenolic compounds were quantified using internal calibration curves of their corresponding standards.^35^ Analysis was performed in duplicate.

### Statistical Analysis

Experiments were performed with three biological replicates (independent experiments) and with two to three technical replicates of each sample. Normal data distribution was verified by the Shapiro–Wilk test. For transepithelial electrical resistance (ΔTEER) and paracellular transport (FITC-dextran) data, each treatment tested in both infected and noninfected cells (Caco-2 and T24) was individually compared to the control incubation (noninfected, no treatment) employing the *t* test. For the UPEC-infected cells (Caco-2 and T24), an additional comparison between each treatment and the incubation with no treatment was also carried out using the *t* test. In the case of Caco-2 cells, the same *t* test was also used to find significant differences between the two effluent treatments (Ef and CB-ef) for both the infected or noninfected states. And, in the case of T24 cells incubated in the presence of urine, the *t* test was used to find significant differences between each treatment and the incubation with no treatment. Significant differences were considered at *p* < 0.05, *p* < 0.01, and *p* < 0.001. Pearson’s correlation coefficients between ΔTEER and (FITC)-dextran values were calculated for the two cell models. The IBM SPSS program (v.28) for Windows was used for data processing.

## Results

### *In Vitro* Effects of Cranberry-Derived Phenolic Metabolites on the Integrity and Functionality of the Intestinal Barrier

Caco-2 cells grown in Transwell inserts under homeostatic conditions, as an intestinal barrier model, were initially infected or noninfected with uropathogenic *Escherichia coli* (UPEC). Then, they were incubated with the simgi effluents (collected before [Ef] or during cranberry feeding [CB-ef]) and the phenolic metabolite 3,4-dihydroxyphenylacetic acid (DOPAC) used as a reference compound for studies of intestinal permeability^32^ (Figure 1). Prior to this experimentation, it was confirmed that the selected concentrations (1:50 [v/v] dilutions for the simgi effluents and 250 μM for DOPAC) did not produce any harmful effects on Caco-2 cells (data not shown).

**Figure 2:**
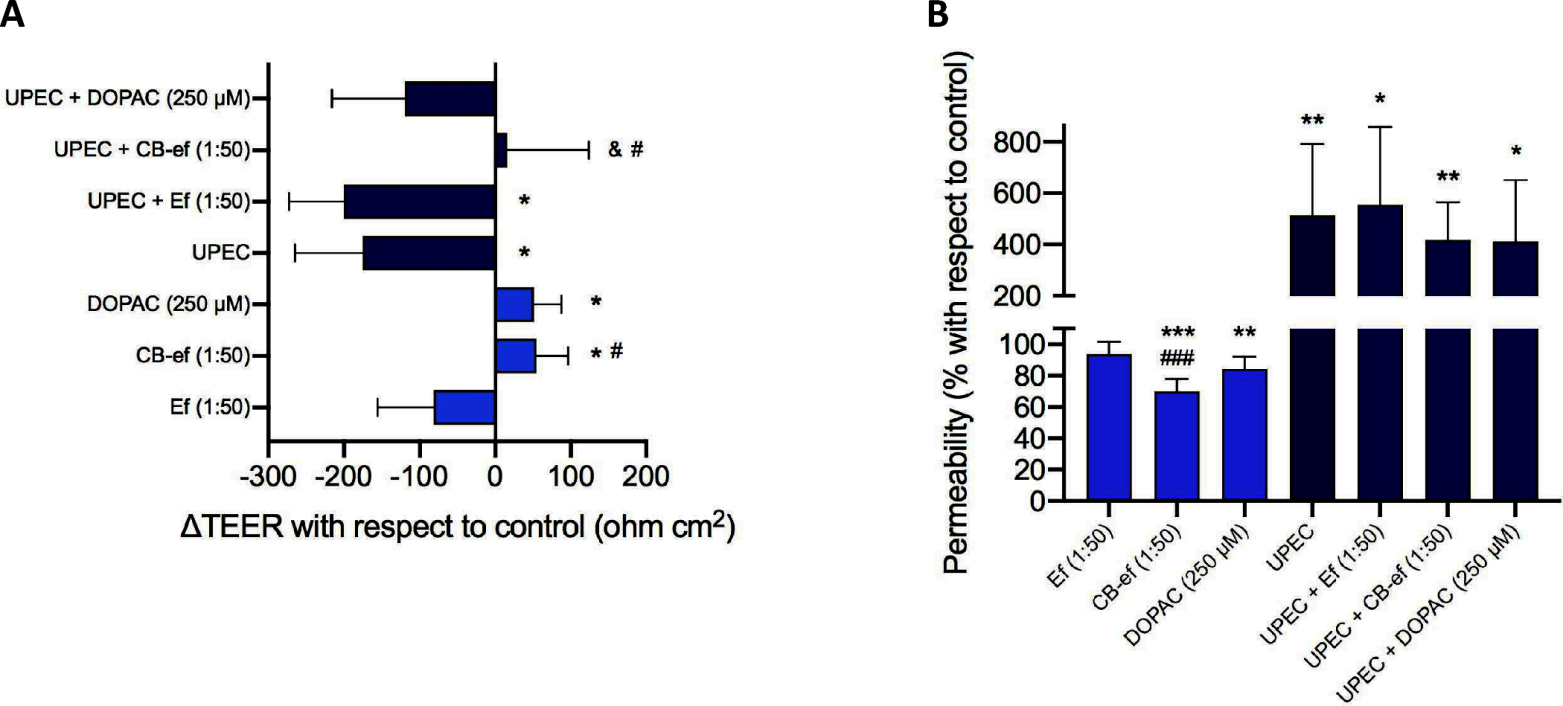
Experiments in Caco-2 cell monolayers grown on Transwell inserts, noninfected or infected with UPEC, and subsequently incubated with simgi effluents (1:50, v/v) collected before (Ef) or during cranberry feeding (CB-ef) and with the phenolic metabolite DOPAC (250 μM). (A) Measurement (mean ± standard deviation) of transepithelial electrical resistance (ΔTEER with respect to control). Values of ΔTEER > 0 are indicative of an improvement in the integrity of the monolayer; values of ΔTEER < 0 are indicative of a loss in the integrity of the monolayer. (B) Measurement (mean ± standard deviation) of paracellular transport with FITC-dextran (% with respect to control). Values <100% are indicative of an improvement in the functionality (i.e., paracellular permeability) of the monolayer; values >100% are indicative of a loss in the functionality of the monolayer. *, **, and *** indicate significant differences (*p* < 0.05, *p* < 0.01, and *p* < 0.001, respectively) with respect to control. ^#^ and ^###^ indicate significant differences (*p* < 0.05 and *p* < 0.001, respectively) with respect to the incubations with effluent medium (Ef) (i.e., CB-ef[1:50] versus Ef[1:50] and UPEC + CB-ef[1:50] versus UPEC + Ef[1:50]). ^&^ indicates significant differences (*p* < 0.05) with respect to the incubations of UPEC-infected cells with no sample (i.e., UPEC + CB-ef[1:50] versus UPEC).

Figure 2 depicts the effects of the selected solutions on transepithelial electrical resistance (TEER), as a means of barrier integrity, and on paracellular transport with FITC-dextran, as a means of barrier functionality, in polarized Caco-2 monolayers not infected or infected with UPEC. As expected, previous infection of the Caco-2 cells with UPEC significantly reduced the transepithelial electrical resistance (ΔTEER < 0 with respect to control, *p* < 0.05) (Figure 2A, Table S3) as well as increased paracellular permeability (FITC-dextran >100 with respect to control, *p* < 0.01) (Figure 2B).

The digestive cranberry (CB-ef, 1:50, v/v) significantly (*p* < 0.05) improved the transepithelial electrical resistance (ΔTEER = 54.5 Ω cm^2^) across the noninfected polarized Caco-2 monolayers despite the adverse effect of the effluent medium itself (Ef, 1:50 v/v) (Figure 2A, Table S3). In the UPEC-infected model, CB-ef treatment (UPEC + CB-ef [1:50]) was found to compensate for the harmful effect due to the bacteria, although differences were not statistically significant with respect to the control (*p* > 0.05), but they were significant with respect to the bacteria treatment (UPEC) (*p* < 0.05) and to the effluent medium (UPEC + Ef [1:50]) (*p* < 0.05) (Figure 2A, Table S3). The same favorable effect (ΔTEER > 0 with respect to control) was also observed for DOPAC (250 μM) in the noninfected model (*p* < 0.05); however, in the UPEC-infected model, the compound could not completely restore the previous damage produced by the bacteria (Figure 2A, Table S3).

**Figure 3:**
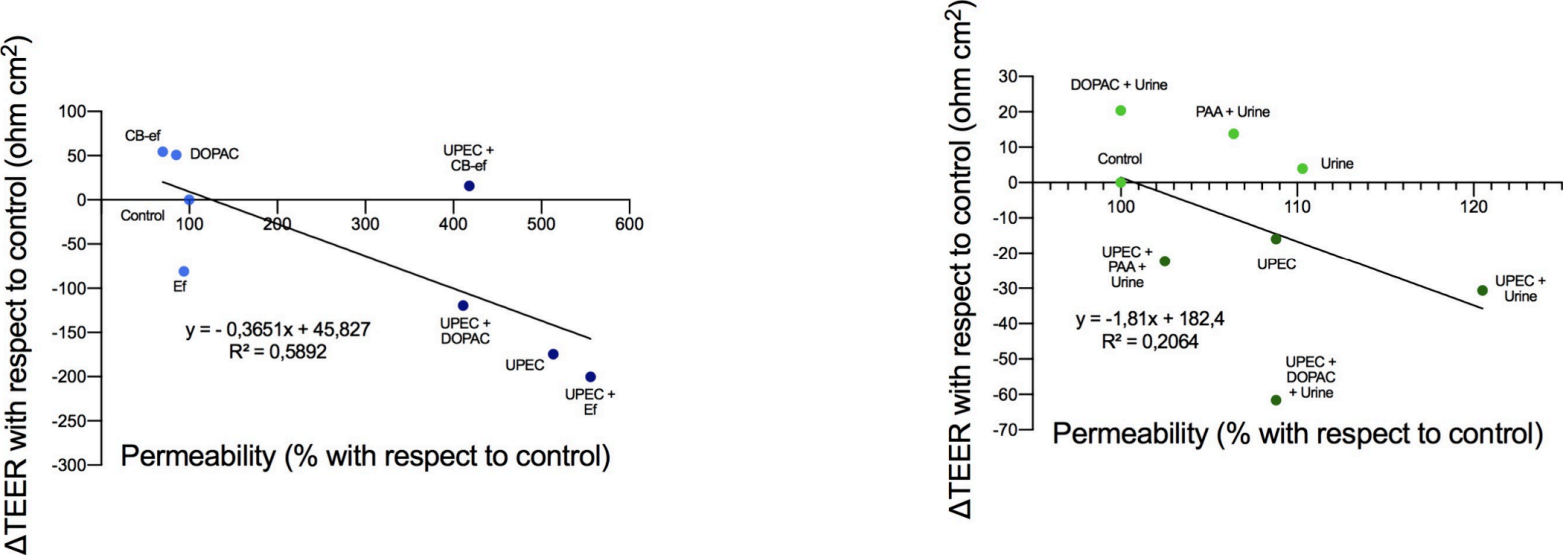
Correlation plots between measurements of barrier integrity (ΔTEER) and functionality (paracellular permeability as FITC-dextran paracellular transport). (A) In Caco-2 cells grown on Transwell inserts, noninfected or infected with UPEC, and subsequently incubated with simgi effluents (1:50, v/v) collected before (Ef) or during cranberry feeding (CB-ef) and with the phenolic metabolite DOPAC (250 μM). (B) In T24 cells grown on Transwell inserts, noninfected or infected with UPEC, and subsequently incubated with urine (1:4, v/v) and with the phenolic metabolites DOPAC (250 μM) and PAA (250 μM) dissolved in urine (1:4, v/v).

With regard to paracellular permeability, values of FITC-dextran paracellular transport corresponding to noninfected Caco-2 cells after treatment with digestive cranberry (CB-ef [1:50]) or DOPAC (250 μM) were significantly lower (by about 30%) with respect to the control (*p* < 0.001 for CB-ef, *p* < 0.01 for DOPAC) (Figure 2B). However, when the cells were previously infected with UPEC, the disruptive damage to paracellular permeability was so great that treatment with CB-ef or DOPAC appeared to have no beneficial effect (Figure 2B). As a comparative summary, Figure 3A illustrates sample distribution in a correlation plot between both variables, i.e., barrier integrity (ΔTEER) and functionality (% of paracellular permeability as FITC-dextran paracellular transport), for both noninfected and UPEC-infected models. A significant negative linear correlation (*p* < 0.05) was found between variables. Higher ΔTEER values (greater barrier integrity) were accompanied by lower levels of paracellular permeability (more accurate barrier functionality), grouping samples into noninfected and UPEC-infected cases (Figure 3A).

**Figure 4:**
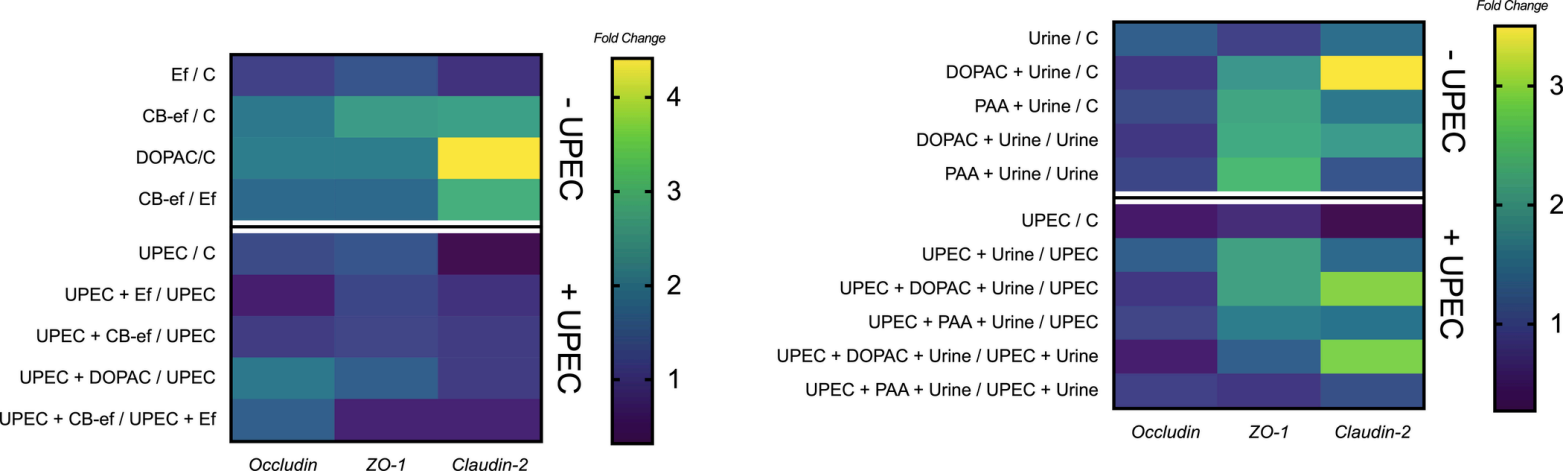
Gene expression of mRNA levels of TJ proteins (occludin, ZO-1, and claudin-2). (A) In Caco-2 cells grown on Transwell inserts, noninfected or infected with UPEC, and subsequently incubated with simgi effluents (1:50, v/v) collected before (Ef) or during cranberry feeding (CB-ef) and with the phenolic metabolite DOPAC (250 μM). (B) In T24 cells grown on Transwell inserts, noninfected or infected with UPEC, and subsequently incubated with urine (1:4, v/v) and with the phenolic metabolites DOPAC (250 μM) and PAA (250 μM) dissolved in urine (1:4, v/v).

The effect of cranberry-derived phenolic metabolites on barrier functionality was also evaluated as the gene expression of TJ proteins (occludin, ZO-1, and claudin-2) in both noninfected and UPEC-infected Caco-2 cell models (Figure 4A, Table S4). For noninfected cells incubated with DOPAC (250 μM) and CB-ef (1:50), increases in mRNA levels were particularly observed for claudin-2 [∼3–4-fold change with respect to control (DOPAC/C and CB-ef/C) and with respect to medium effluent (CB-ef/Ef)] but to a lower extent for occludin and ZO-1 (∼2–3-fold change with respect to control (DOPAC/C and CB-ef/C)] (Figure 4A). In contrast, the effluent Ef seemed to promote a slightly downregulated effect with respect to control [<1-fold change (Ef/C)], especially for claudin-2 (Figure 4A). Similar downregulated effects in TJ protein expression were observed when cells were infected with UPEC (UPEC/C, Figure 4A). Although, in general, changes in the UPEC-infected model were much less notable than those in the noninfected model, it is worth noting that there was a modest recovery in the mRNA levels of occludin when infected cells were incubated with DOPAC or CB-ef (∼1–2-fold change with respect to UPEC [UPEC + DOPAC/UPEC and UPEC + CB-ef/UPEC]) and in ZO-1 (∼1–2-fold change with respect to UPEC [UPEC + DOPAC/UPEC]) (Figure 4A). No changes in claudin-2 mRNA levels were found when UPEC-infected cells were incubated with cranberry-derived phenolic metabolites (DOPAC or CB-ef; Figure 4A).

### *In Vitro* Effects of Cranberry-Derived Phenolic Metabolites on the Integrity and Functionality of the Urinary Barrier

As a urinary barrier model, T24 cells grown in Transwell inserts under homeostatic conditions were initially infected or not infected with UPEC and then incubated with 3,4-dihydroxyphenylacetic acid (DOPAC) and phenylacetic acid (PAA) (Figure 1). Moreover, to simulate *in vivo* conditions as closely as possible, compounds should be dissolved in the urine. Therefore, the cytotoxicity of the urine sample collected for this aim was initially tested with 1:1 (no dilution), 1:2, and 1:4 v/v dilutions. Toxic effects (cell viability <80%) against T24 cells were found for nondiluted urine and for 1:2 (v/v) diluted urine but not for the 1:4 (v/v) dilution (data not shown). Based on this, the phenolic metabolites DOPAC and PAA dissolved in urine (1:4, v/v) at a physiological concentration of 250 μM were tested for their toxicity against T24 cells. No harmful effects were observed (data not shown), in line with previous experiments using the cell culture medium as solvent.^22^

**Figure 5:**
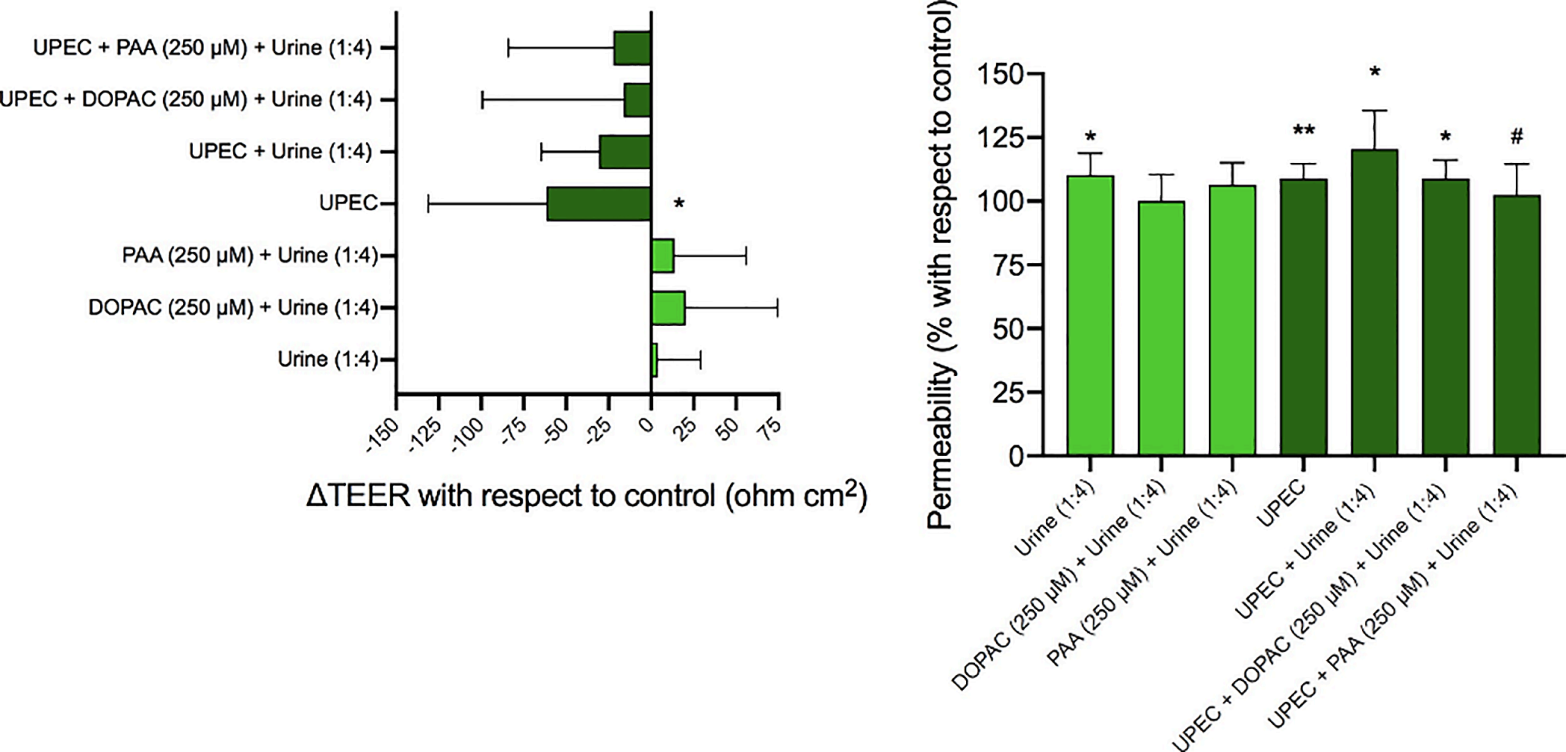
Experiments in T24 cell monolayers grown on Transwell inserts, noninfected or infected with UPEC, and subsequently incubated with urine (1:4, v/v) and with the phenolic metabolites DOPAC (250 μM) and PAA (250 μM) dissolved in urine (1:4, v/v). (A) Measurement (mean ± standard deviation) of transepithelial electrical resistance (ΔTEER with respect to control). Values of ΔTEER > 0 are indicative of an improvement in the integrity of the monolayer; values of ΔTEER < 0 are indicative of a loss in the integrity of the monolayer. (B) Measurement (mean ± standard deviation) of paracellular transport with FITC-dextran (% with respect to control). Values <100% are indicative of an improvement in the functionality (i.e., paracellular permeability) of the monolayer; values >100% are indicative of a loss in the functionality of the monolayer. * and ** indicate significant differences (*p* < 0.05 and *p* < 0.01, respectively) with respect to control. ^#^ indicates significant differences (*p* < 0.05) with respect to the incubation of UPEC-infected cells with urine (1:4, v/v) (i.e., UPEC + PAA[250 μM] + urine[1:4] versus UPEC + urine [1:4]).

As seen for Caco-2 cells, UPEC infection of the T24 cells significantly reduced the transepithelial electrical resistance (ΔTEER < 0 with respect to control, *p* < 0.05) (Figure 5A, Table S3) as well as increased paracellular permeability (FITC-dextran >100 with respect to control, *p* < 0.01) (Figure 5B). The urine (1:4, v/v) dilution used as solvent seemed not to affect the transepithelial electrical resistance (ΔTEER ∼0 with respect to control) (Figure 5A, Table S3), although a significant increase in paracellular permeability (FITC-dextran >100 with respect to control, *p* < 0.05) was observed (Figure 5B).

In both noninfected and UPEC-infected urinary barrier models, the phenolic metabolites DOPAC and PAA [250 μM in (1:4, v/v) urine] slightly improved the transepithelial electrical resistance in comparison to the respective controls (urine [1:4] and UPEC + urine [1:4], respectively), but nonsignificant differences (*p* > 0.05) were found in any case (Figure 5A, Table S3). Similarly, slight but insignificant (*p* > 0.05) improvements associated with DOPAC and PAA treatments were observed in paracellular permeability in both noninfected and UPEC-infected models despite the adverse effect of the urine (1:4) and UPEC + urine (1:4) mixtures (Figure 5B). Only PAA treatment of UPEC-infected cells (UPEC + PAA[250 μM] + urine[1:4]) led to a significant decrease (*p* < 0.05) with respect to the incubations of UPEC-infected cells with urine (UPEC + urine [1:4]) (Figure 5B). In a correlation plot between measurements of barrier integrity (ΔTEER) and functionality (FITC-dextran paracellular transport), samples from both noninfected and UPEC-infected models were widely distributed, and no significant linear correlation (*p* > 0.05) was found between these two variables (Figure 3B).

With regard to TJ protein gene expression, claudin-2 showed the biggest changes with respect to control as a consequence of the different treatments (up to 3.5-fold change), whereas almost no changes in mRNA levels for occludin were observed (Figure 4B, Table S4). Urine (1:4, v/v) itself showed slightly upregulated effects for TJ protein gene expression (1–2-fold change with respect to control [urine/C]), whereas UPEC infection seemed to suppress it (<1-fold change [UPEC/C]) (Figure 4B). For noninfected T24 cells, incubation with DOPAC (250 μM) increased mRNA levels of claudin-2 and ZO-1 (3.5- and ∼2-fold change with respect to control, respectively [DOPAC + urine/C]). PAA (250 μM) also induced upregulation, but in this case, ZO-1 was more sensitive than claudin-2 (∼1.5- and >2-fold change with respect to control, respectively [PAA + urine/C]) (Figure 4B). These upregulating effects for TJ protein gene expression associated with DOPAC and PAA were also observed in UPEC-infected cells but to a lower extent in general (Figure 4B), which were in line with the trends observed for paracellular permeability (Figure 5B).

## Discussion

In antiquity, cranberry was used by American natives for the treatment of wounds and infections. Nowadays, the initial empirical knowledge has been translated into scientific evidence by numerous *in vitro*, *ex vivo*, and *in vivo* studies that support the medical recommendation of cranberry consumption for prophylaxis against UTI. However, this general recommendation is not without some controversy due to the large interindividual variability observed in different studies (see Xia et al.^36^ and Valente et al.^37^ for recent meta-analyses). Indeed, a full clarification of the action mechanisms of cranberry against UTI would result in a better understanding of the differences between individuals/population groups in response to its consumption and the design of more effective products/treatments against these infections. Recent studies have shed light on the antiadherence activity against bacteria at the urothelial level derived from cranberry consumption;^1^ however, other mechanisms that might be jointly implicated at both urinary and intestinal barriers needed further investigation. Therefore, the present study highlights some new findings about the protective effects against UTI/UPEC of cranberry-derived phenolic metabolites in relation to the integrity and functionality of both barriers: the intestinal barrier, related to which there was a previous pioneer study,^25^ and the urinary barrier, for which we have found no previous references.

It was to be expected that UPEC infection would trigger severe damage to mucosal epithelia in both the intestinal and urinary tracts because of their physiological similarities. Both share common core functions, as they offer a physical barrier assisted by the tight junctions (TJ) formed by neighboring epithelial cells. TJ mainly consist of two functional protein categories: integral transmembrane proteins that form a network between adjacent cell membranes and peripheral membrane, or plaque proteins that act as bridges to connect integral membrane proteins to the actin cytoskeleton and to other signaling proteins.^38^ At the intestinal level, integral transmembrane proteins are occludin, claudins (mainly claudin-2, -10, and -15 isoforms), junctional adhesion molecules (JAMs), and tricellulin, whereas peripheral membrane adaptor proteins include ZO-1, ZO-2, and ZO-3.^38^ At the urinary level, TJ comprise ZO-1, occludin, and claudin-4, -8, and -12.^6^ In our study, permeability results showed that UPEC infection in the absence of cranberry-derived phenolic metabolites led to a significant loss of barrier integrity (measured as ΔTEER) in both Caco-2 and T24 models, more pronounced at the intestinal level (Figures 2A and 5A). Accordingly, paracellular permeability (measured as FITC-dextran) markedly increased, notably for the intestinal model (Figures 2B and 5B), which was attributed to the T24 cells’ greater resistance to UPEC strains (i.e., *E. coli* ATCC 53503) in comparison to Caco-2 cells, as bladder cells and UPEC inhabit the urinary environment. In addition, it should be noted that Caco-2 cell monolayers spontaneously differentiated into a polarized cell layer with apical microvilli upon reaching confluence, which made them more sensitive to bacterial infection. On the other hand, our results agreed with a previous study that found loss in paracellular permeability together with secretion of cytokines in a model of UPEC infection *in vitro* using canine bladder mucosa mounted in Ussing chambers.^39^ These authors hypothesized that the paracellular permeability defect might be associated with the failure of umbrella cell tight junction formation and umbrella cell sloughing.^39^ In a more recent study, Tian et al.^10^ found suppression of TJ protein expression (i.e., ZO-1 and occludin) in UPEC-infected monolayers of bladder epithelial cells. Again, our results showed that the expression of TJ protein genes was downregulated in UPEC-infected T24 cells, albeit to a small extent (Figure 3B).

With regard to cranberry-derived phenolic metabolites and their protection against UTI, our results from the Caco-2 cell model proved that there were improvements in intestinal barrier integrity and functionality after treatment with a cranberry-derived digestive fluid (CB-ef) (Figures 2 and 4A). This favorable effect was attributed to the CB-Ef content in phenolic metabolites (135 mg/L) in comparison to that of the medium itself (Ef, 38.1 mg/L) (Table 1). As expected, the cranberry-derived digestive fluid (CB-ef) was particularly rich in benzoic acids (i.e., benzoic and vanillic acids), phenylpropanoic acids (i.e., 3-[4′-hydroxyphenyl]propanoic acid), and phenylacetic acids (i.e., 3-hydroxyphenylacetic acid and phenylacetic acid), which derived from the degradation of cranberry polyphenols.^16^ These main groups of phenolic metabolites were also found in other cranberry-derived fluids using other digestion procedures in previous studies.^15,40^ Differences in individual phenolic compounds among studies were attributed to the intrinsic variability in human fecal microbiota and the analytical methodologies used, apart from the fact that, in previous studies, cranberry extracts were not subjected to gastrointestinal digestion before colonic fermentation.

From the first evidence reported by Faggian et al.,^25^ the findings of our study go further in demonstrating the protective effects of cranberry-derived phenolic metabolites in relation to the integrity of the intestinal barrier. First, in a homeostatic or healthy model, our results confirmed the active role of gut-derived cranberry metabolites in maintaining the integrity of the intestinal epithelium as it was found that CB-ef improved barrier integrity in Caco-2 cells (ΔTEER > 0 with respect to control, Figure 2A). This effect was not found when cranberry products were directly tested, probably because they were not subjected to previous intestinal digestion and cranberry-phenolic metabolites were not formed.^25^ Second, our study found that treatment with CB-ef in UPEC-infected Caco-2 cells completely restored the harmful effect due to the bacteria (Figure 2A), whereas when cells were treated with different cranberry products without any previous intestinal digestion,^25^ the recovery of TEER values was partial for only one of the cranberry products tested. Similarly to the paper of Faggian et al.,^25^ our study also evaluated changes in various barrier functionality parameters (FITC-dextran and gene expression of the TJ proteins, Figures 2B and 4A), in which FITC-dextran correlated well with changes observed in barrier integrity (TEER) (Figure 3A). In a more extended way, our study contributes new evidence that supports the role of dietary polyphenols in modulating intestinal permeability, an issue of current interest as it has been associated with several pathological or dysfunctional conditions.^8^ Several recent studies have demonstrated protective effects on intestinal paracellular permeability of polyphenol-rich products using different cellular models in inflammatory or homeostatic conditions.^32,33,41,42^

In relation to DOPAC (3,4-dihydroxyphenylacetic acid) as reference compound, our results agreed with previous studies that reported improvement of the integrity of the intestinal barrier using Caco-2 cells^32,33,43,44^ or HT-29 cells^41^ as an *in vitro* model. On the other hand, the metabolite DOPAC (250 μM) seemed to exert slightly lower protective effects than the whole mixture of cranberry-derived phenolic metabolites (Figure 2), even bearing in mind that the total phenolic molar concentration of CB-ef (1:50, v/v) was calculated as 19.5 μM (data not shown). This was explained in terms of different activity among phenolic structures and/or of the presence of other active nonidentified phenolic compounds in the cranberry-derived digested fluid as well as in potential synergistic effects among them.

In terms of urinary barrier (T24 cells model), both DOPAC and PAA (phenylacetic acid), at a concentration of 250 μM, appeared to improve barrier integrity (measured as ΔTEER) and functionality (measured as paracellular transport with FITC-dextran and as gene expression of the TJ proteins occludin, ZO-1, and claudin-2), although differences were not significant (*p* > 0.05) in most cases (Figures 4B and 5). In an intervention study, it was found that the total amounts of these compounds excreted in urine 24 h after the intake of cranberry juice (787 mg of polyphenols) were 1.60 and 14.7 μM for DOPAC acid and PAA, respectively.^45^ Therefore, we cannot reject the possibility of *in vivo* improvements in urinary barrier integrity and functionality after the intake of cranberry products as additive (and even synergistic) effects among all of the cranberry-derived phenolic metabolites present in urine. To the best of our knowledge, this is the first study to evaluate the potential impact of cranberry-derived phenolic metabolites on the integrity and functionality of the urinary barrier.

In conclusion, the results of this paper demonstrate the joint effects of cranberry-derived phenolic metabolites on the intestinal and urothelial epithelia in two physiological conditions: a healthy state and a uropathogenic bacteria-infected state. Our findings contribute new evidence and provide novel clues enhancing the knowledge of the mechanisms underlying the protective effects of the consumption of cranberry against UTI. In particular, we showed that cranberry-derived metabolites improve the integrity and paracellular permeability of both intestinal and urinary barriers *in vitro*. The findings also revealed that the expression levels of TJ proteins play a critical role in regulating the function of both barriers and in repairing their disruption due to cell injury and harmful effects of bacterial infection. However, the protective action of cranberry-derived phenolic metabolites at both intestinal and urinary epithelia seemed to be more effective in the healthy state than in the UPEC-infected state. In short, cranberry polyphenols might ameliorate UTI via improving integrity and functionality of intestinal and urinary barriers, but at both levels, the mechanisms of action (inhibition of bacterial adhesion, reinforcement of barrier functionality, interaction with the microbiota, modulation of the inflammatory response, among others) must be understood holistically and dynamically. Global experiments covering all of these mechanisms should be considered in the future.

## Supporting Information Available

Quantity (ng/μL) and quality assurance (ratio 260/280) of extracted RNA (Table S1); primer sequences and melting temperature (*T*_melting_) used for TJ protein quantification by qRT-PCR (Table S2); data (mean ± standard deviation) of transepithelial electrical resistance (TEER) (Ω cm^2^) from samples and control, before (*t* = 0 h) and after treatments (*t* = 4 h), and for Caco-2 and T24 cells; calculation of ΔTEER with respect to control is also included (Table S3); and PCR data of TJ proteins (occludin, Z0–1, and claudin-2) expression as fold change (mean ± standard deviation) with respect to the noninfected control (C), the effluent Ef, the infected control (UPEC), the effluent Ef in the infected model (UPEC + Ef), or urine (Urine) (Table S4) (PDF)

## Abbreviations Used

CB-ef: effluent from the simgi after 15 days of cranberry feeding
DOPAC: 3,4-dihydroxyphenylacetic acid
Ef: effluent from the simgi before cranberry feeding
FITC: fluorescein isothiocyanate
PAA: phenylacetic acid
TEER: transepithelial electrical resistance
TJ: tight junctions
UPEC: uropathogenic *Escherichia coli*
UTI: urinary tract infections
ZO-1: zonula occludens-1
