# Supplementary material for: Cranberry Polyphenols and Prevention against Urinary Tract Infections: New Findings Related to the Integrity and Functionality of Intestinal and Urinary Barriers
Source: J Agric Food Chem. 2024 Apr 23;72(18):10328–38. doi: 10.1021/acs.jafc.3c07169 (PMC11082924; doi:10.1021/acs.jafc.3c07169)
Supplement: Supplementary file 1 — jf3c07169_si_001.pdf [file jf3c07169_si_001.pdf]

Cranberry Polyphenols and Prevention against Urinary Tract Infections: New Findings Related to the Integrity and Functionality of Intestinal and Urinary Barriers

González de Llano, Dolores; Roldán, Mikel; Taladrid, Diego; Relaño de la Guía, Edgard; Moreno-Arribas, M. Victoria; Bartolomé, Begoña

SUPPLEMENTARY MATERIAL

Table S1. Quantity (ng/µL) and quality assurance (Ratio 260/280) of extracted RNA.

Caco-2 cells					T24 cells				
		Independent assays					Independent assays		
		1	2	3			1	2	3
Control	RNA (ng/uL)	418	689	1111	Control	RNA (ng/uL)	304	555	465
	A260/A280	2.1	2.1	2.1		A260/A280	2.1	2.1	2.1
CB-ef (1:50)	RNA (ng/uL)	565	548	197	Urine (1:4)	RNA (ng/uL)	162	373	265
	A260/A280	2.1	2.1	2.1		A260/A280	2.1	2.1	2.1
Ef (1:50)	RNA (ng/uL)	426	769	383	DOPAC (250µ) + Urine (1:4)	RNA (ng/uL)	156	324	274
	A260/A281	2.1	2.1	2.1		A260/A281	2.1	2.1	2.1
DOPAC (250µ)	RNA (ng/uL)	550	369	443	PA (250µ) + Urine (1:4)	RNA (ng/uL)	205	300	173
	A260/A281	2.1	2.1	2.1		A260/A281	2.1	2.1	2.1
UPEC	RNA (ng/uL)	729	682	403	UPEC	RNA (ng/uL)	241	730	291
	A260/A282	2.1	2.1	2.1		A260/A282	2.1	2.1	2.1
UPEC + CB-ef (1:50)	RNA (ng/uL)	480	849	533	UPEC + Urine (1:4)	RNA (ng/uL)	175	253	160
	A260/A282	2.1	2.1	2.0		A260/A282	2.1	2.1	2.1
UPEC + Ef (1:50)	RNA (ng/uL)	746	970	620	UPEC + DOPAC (250µ) + Urine (1:4)	RNA (ng/uL)	172	247	310
	A260/A283	2.1	2.1	2.0		A260/A283	2.1	2.1	2.1
UPEC + DOPAC (250µ)	RNA (ng/uL)	581	462	343	UPEC + PA (250µ) + Urine (1:4)	RNA (ng/uL)	183	297	335
	A260/A283	2.1	2.1	2.1		A260/A283	2.1	2.1	2.1

Table S2. Primer sequences and melting temperature ($T_{melting}$) used for TJ protein quantification by qRT-PCR.

**Target genes**	**Oligonucleotide sequences**	**$T_{melting}$ (ºC)**
Occludin	*Forward:* 5′-ATGAGACAGACTACACAACTGG-3′<br>*Reverse:* 5′-TTGTATTCATCAGCAGCAGC-3′	54.1
ZO-1	*Forward:* 5′-GGTGAAGTGAAGACAATG-3′<br>*Reverse:* 5′-GGTAATATGGTGAAGTTAGAG-3′	50.6
Claudin-2	*Forward*: 5′-ATTGCTTCTTGATGCTTA-3′<br>*Reverse*: 5′-GAGAGGTCTAGGATATGG-3′	47.7
GA3PDH*	*Forward*: 5′-TGCACCACCAACTGCTTAGC-3′<br>*Reverse*: 5′-GGCATGGACTGTGGTCATGAG-3′	59.4

* Housekeeping gene. The mRNA levels of each protein in the different samples were normalized against GADPH and expressed as the fold increase with respect to the control, using the E-ΔΔCT method.

Table S3. Data (mean ± standard deviation) of transepithelial electrical resistance (TEER) (ohm cm^2) from samples and control, before (t=0h) and after treatments (t=4h), and for Caco-2 and T24 cells. Calculation of ΔTEER with respect to control is also included.

Caco-2 cells (differentiated monolayers grown on Transwell® inserts, 21 d)

	TEER (t= 0h) (ohm cm^2)	TEER (t=4h) (ohm cm^2)	ΔTEER with respect to control (ohm cm^2)*
**Control**	1940 ± 231	1827 ± 84	
**Ef (1:50)**	1945 ± 323	1751 ± 99	-80.9 ± 78.2
**CB-ef (1:50)**	1894 ± 314	1835 ± 125	54.5 ± 41.8
**DOPAC (250 µM)**	1848 ± 81	1786 ± 102	51.0 ± 36.9
**UPEC**	1551 ± 225	1259 ± 258	-179 ± 91
**UPEC + Ef (1:50)**	1496 ± 238	1182 ± 145	-201 ± 72
**UPEC + CB-ef (1:50)**	1472 ± 258	1375 ± 287	16.0 ± 99.4
**UPEC + DOPAC (250 µM)**	1657 ± 323	1424 ± 310	-119 ± 92

T24 cells (proliferative monolayers grown on Transwell® inserts, 3 d)

	TEER (t= 0h) (ohm cm^2)	TEER (t=4h) (ohm cm^2)	ΔTEER with respect to control (ohm cm^2)*
**Control**	648 ± 51.9	694 ± 49.6	
**Urine (1:4)**	629 ± 60.8	679 ± 21.6	4.10 ± 23.3
**DOPAC (250 µM) + Urine (1:4)**	612 ± 75.3	678 ± 54.0	20.4 ± 56.4
**PAA (250 µM) + Urine (1:4)**	630 ± 61.1	690 ± 42.9	13.8 ± 47.8
**UPEC**	652 ± 61.3	637 ± 37.1	-61.6 ± 69.6
**UPEC + Urine (1:4)**	642 ± 62.8	658 ± 37.5	-30.7 ± 30.5
**UPEC + DOPAC (250 µM) + Urine (1:4)**	628 ± 73.1	659 ± 41.5	-16.1 ± 83.3
**UPEC + PAA (250 µM) + Urine (1:4)**	650 ± 61.5	674 ± 29.9	-22.3 ± 68.2

*$\Delta TEER_{sample}$ (with respect to the control) = ($TEER_{(t=4h)sample}$ – $TEER_{(t=0h)sample}$) – ($TEER_{(t=4h)control}$ – $TEER_{(t=0h)control}$)

Table S4. PCR data of TJ proteins (Occludin, Z0-1, and claudin-2) expression as Fold Change (mean ± standard deviation) with respect to the noninfected control (C), the effluent Ef, the infected control (UPEC), the effluent Ef in the infected model (UPEC + Ef) or urine (Urine).

Caco-2 cells (differentiated monolayers grown on Transwell® inserts, 21 d)

	Occludin	ZO-1	Claudin-2
**Ef / C**	1.13 ± 0.28	1.37 ± 0.36	0.91 ± 0.10
**CB-ef / C**	2.01 ± 0.60	2.58 ± 0.28	2.67 ± 0.82
**DOPAC / C**	2.05 ± 0.71	2.03 ± 0.46	4.41 ± 1.42
**CB-ef / Ef**	1.75 ± 0.24	1.67 ± 0.38	2.93 ± 0.74
**UPEC / C**	1.29 ± 0.29	1.39 ± 0.33	0.32 ± 0.05
**UPEC + Ef / UPEC**	0.67 ± 0.06	1.19 ± 0.25	0.90 ± 0.09
**UPEC + CB-ef / UPEC**	1.07 ± 0.14	1.17 ± 0.27	1.06 ± 0.30
**UPEC + DOPAC / UPEC**	1.96 ± 0.39	1.54 ± 0.23	1.07 ± 0.12
**UPEC + CB-ef / UPEC + Ef**	1.59 ± 0.28	0.72 ± 0.18	0.71 ± 0.31

T24 cells (proliferative monolayers grown on Transwell® inserts, 3 d)

	Occludin	ZO-1	Claudin-2
**Urine / C**	1.25 ± 0.31	0.90 ± 0.13	1.46 ± 0.24
**DOPA + Urine / C**	0.78 ± 0.27	1.99 ± 0.43	3.50 ± 0.22
**PAA + Urine / C**	1.03 ± 0.26	2.22 ± 0.16	1.60 ± 0.20
**DOPAC + Urine / Urine**	0.81 ± 0.20	2.27 ± 0.50	2.03 ± 0.59
**PAA + Urine / Urine**	0.96 ± 0.28	2.48 ± 0.17	1.12 ± 0.04
**UPEC / C**	0.48 ± 0.19	0.68 ± 0.18	0.27 ± 0.10
**UPEC + Urine / UPEC**	1.23 ± 0.16	2.17 ± 0.38	1.35 ± 0.24
**UPEC + DOPAC + Urine / UPEC**	0.78 ± 0.12	2.16 ± 0.28	2.94 ± 0.60
**UPEC + PAA+ Urine / UPEC**	0.94 ± 0.19	1.65 ± 0.44	1.29 ± 0.01
**UPEC + DOPAC + Urine /UPEC + Urine**	0.54 ± 0.13	1.23 ± 0.23	2.87 ± 0.21
**UPEC + PAA + Urine / UPEC + Urine**	0.88 ± 0.12	0.77 ± 0.10	1.06 ± 0.54
